# Modulation of TLR3/TLR4 inflammatory signaling by the GABA_B_ receptor agonist baclofen in glia and immune cells: relevance to therapeutic effects in multiple sclerosis

**DOI:** 10.3389/fncel.2015.00284

**Published:** 2015-07-28

**Authors:** Tadhg Crowley, John-Mark Fitzpatrick, Teun Kuijper, John F. Cryan, Orna O’Toole, Olivia F. O’Leary, Eric J. Downer

**Affiliations:** ^1^Department of Anatomy and Neuroscience, University College CorkCork, Ireland; ^2^Alimentary Pharmabiotic Centre, University College CorkCork, Ireland; ^3^Mercy University HospitalCork, Ireland; ^4^School of Medicine, Discipline of Physiology, Trinity Biomedical Sciences Institute, Trinity CollegeDublin, Ireland

**Keywords:** glia, TLR, multiple sclerosis, GABA, baclofen, inflammation, innate immunity

## Abstract

The GABA_B_ receptor agonist, baclofen, is used to treat muscle tightness and cramping caused by spasticity in a number of disorders including multiple sclerosis (MS), but its precise mechanism of action is unknown. Neuroinflammation drives the central pathology in MS and is mediated by both immunoreactive glial cells and invading lymphocytes. Furthermore, a body of data indicates that the Toll-like receptor (TLR) family of innate immune receptors is implicated in MS progression. In the present study we investigated whether modulation of GABA_B_ receptors using baclofen can exert anti-inflammatory effects by targeting TLR3 and(or) TLR4-induced inflammatory signaling in murine glial cells and human peripheral blood mononuclear cells (PBMCs) isolated from healthy control individuals and patients with the relapse-remitting (RR) form of MS. TLR3 and TLR4 stimulation promoted the nuclear sequestration of NF-κB and pro-inflammatory cytokine expression in murine glia, while TLR4, but not TLR3, promoted pro-inflammatory cytokine expression in PBMCs isolated from both healthy donors and RR-MS patients. Importantly, this effect was exacerbated in RR-MS patient immune cells. We present further evidence that baclofen dose-dependently attenuated TLR3- and TLR4-induced inflammatory signaling in primary glial cells. Pre-exposure of PBMCs isolated from healthy donors to baclofen attenuated TLR4-induced TNF-α expression, but did not affect TLR4-induced TNF-α expression in RR-MS patient PBMCs. Interestingly, mRNA expression of the GABA_B_ receptor was reduced in PBMCs from RR-MS donors when compared to healthy controls, an effect that might contribute to the differential sensitivity to baclofen seen in healthy and RR-MS patient cells. Overall these findings indicate that baclofen differentially regulates TLR3 and TLR4 signaling in glia and immune cells, and offers insight on the role of baclofen in the treatment of neuroinflammatory disease states including MS.

## Introduction

Autoimmunity drives the development of multiple sclerosis (MS), involving central nervous system (CNS) infiltration of immune cells, myelin degradation, reactive changes in glia and axonal loss (Compston and Coles, 2002). The innate immune system has received much interest as having a defined role in the progression and/or etiology of MS (O’Brien et al., 2008; Downer, 2011). Innate immunity is regulated by complex mechanisms involving pattern-recognition receptors (PRRs) that recognize molecular signatures of microbes. Intracellular signaling triggered by PRRs leads to transcriptional expression of inflammatory mediators that coordinate the elimination of pathogens (Akira et al., 2006). If left unchecked, or not tightly regulated, dysregulation of this system can lead to conditions such as sepsis and autoimmunity (Lehnardt, 2010).

Toll-like receptors (TLRs) belong to the family of signaling PRRs (Jeannin et al., 2008) that initiate innate immune reactions by activating transcription factors such as nuclear factor (NF)-κB, in addition to inducing the expression of interferons (IFNs) and cytokines. Thus far, 12 functional TLRs have been identified in mice, and 10 in humans (O’Neill, 2004; Kawai and Akira, 2006). TLRs are localized in endosomal compartments (including TLR3, TLR7, TLR8, TLR9), or are cell membrane-bound (as with all other TLRs; O’Neill, 2004), and couple to specific signaling cascades, inducing gene transcription and controlling immune processes, with this specificity reliant on the TLR adaptor proteins recruited (O’Neill, 2004). All TLRs (with the exception of TLR3), recruit the adaptor myeloid differentiation factor 88 (MyD88; Medzhitov et al., 1998). TLR3 (and TLR4) induces MyD88-independent signaling to couple to NF-κB via Toll-Interleukin-1 Receptor (TIR)-domain-containing adaptor-inducing IFN-β (TRIF) protein. TLRs are key players in CNS diseases, and with respect to MS, key roles of TLRs have been shown in murine models of MS (Touil et al., 2006), while the expression of TLRs characterized on immune cells and CNS glia and neurons (Nishimura and Naito, 2005).

Gamma amino butyric acid (GABA) is the major inhibitory amino acid neurotransmitter in the brain (Cobb et al., 1995). GABA exerts its effects through the ionotropic receptors, GABA_A_/GABA_A-ρ_ and the metabotropic receptor GABA_B_ (Bormann, 2000; Campagna-Slater and Weaver, 2007; Pinard et al., 2010), and GABA receptor transcripts are present in neurons, glia (Lee et al., 2011; Vélez-Fort et al., 2012), and immune cells (Bhat et al., 2010). There is a growing body of evidence pointing to the key role of GABA receptors in neuroinflammation (Ziegler et al., 1980; Tyagi et al., 2015), and recent reports has identified that modulation of the GABAergic system occurs in MS (Han et al., 2008; Carmans et al., 2013). Indeed, GABA insufficiency has been identified in MS patients (Demakova et al., 2003), while intrathecal administration of the GABA_B_ receptor agonist, baclofen, is used as a treatment strategy to control spasticity in patients with MS (Gunnarsson and Samuelsson, 2015). Furthermore, enhancing endogenous GABA via administration of the GABA-degrading enzyme GABA-transaminase (GABA-T), has been shown to be protective in an experimental autoimmune encephalomyelitis (EAE) mouse model (Carmans et al., 2013), indicating that increasing endogenous GABA also has therapeutic potential in the murine model of MS. In support of these *in vivo* findings, *in vitro* studies have shown that baclofen reduces TLR4-induced release of pro-inflammatory cytokines from primary murine microglia (Kuhn et al., 2004), indicating that cross talk may exist between the GABAergic and TLR systems, with relevance to inflammatory signaling events.

GABA_B_ receptors are metabotropic G_i_/G_o_-coupled receptors (Padgett and Slesinger, 2010) which are distributed throughout the CNS and periphery (Ong and Kerr, 1990; Hyland and Cryan, 2010). GABA_B_ receptors can function to regulate ion channels (activate K^+^ and inhibit Ca^2+^ channels) and cellular signaling (adenylate cyclase, MAPK; Kornau, 2006; Jiang et al., 2012), limit the release of neurotransmitters (GABA, glutamate; Pinard et al., 2010; Gassmann and Bettler, 2012), and dampen depolarisation induced by excitatory neurotransmitters. GABA_B_ receptors are implicated in a variety of neurodegenerative, neuroinflammatory, and pathophysiological disorders including depression, spasticity, pain, and schizophrenia (Reyes-García et al., 2007; Bjurstöm et al., 2008; Garcia-Oscos et al., 2012). Given previous reports linking TLR’s and the GABA_B_ receptor subtype with neuroinflammation, particularly with relevance to MS pathogenesis, we sought to explore the impact of baclofen (the GABA_B_ receptor agonist) on TLR3 and TLR4-induced inflammatory signaling both centrally and in the periphery, using murine glial cells and human peripheral blood mononuclear cells (PBMCs) isolated from healthy individuals and newly-diagnosed patients with the relapsing-remitting (RR) form of MS patients. This study identifies baclofen as a differential regulator of TLR3 and TLR4 signaling in glia and immune cells, and offers insight on the role of baclofen in regulating the innate immune response in cellular pathology associated with MS.

## Materials and Methods

### Preparation of Primary Mixed Glial Cultures and Treatments

Primary mixed glia were prepared from the whole brain of 1-day-old C57/BL6 mice and plated (at 5 × 10^5^ cells/ml) as previously described (Downer et al., 2010). All experiments were performed under a license issued by the Health Products Regulatory Authority (Ireland) and in accordance with the European Directive 86/609/EEC and the guidelines laid down by the Animal Experimentation Ethics Committee of University College Cork. After 14 days in culture, mixed glia were treated with the TLR4 ligand LPS (100 ng/ml; Sigma–Aldrich, UK), the TLR3 ligand Poly(I:C) (10 μg/ml; InvivoGen, France) or vehicle (sterile H_2_O) for timepoints ranging from 10 min–24 h. In a second series of experiments, mixed glia were exposed to the GABA_B_ agonist baclofen (10, 30, and 100 μM; Sigma–Aldrich, Germany) for 30 min prior to LPS (100 ng/ml; 30 min and 24 h) or Poly(I:C) (10 μg/ml; 6 h and 24 h) exposure.

### Patients and Blood Samples

Healthy donors and RR-MS patients attending outpatient clinics at the Mercy University Hospital, Cork, were recruited for this study. Written informed consent was obtained from each participant and the study received ethical approval from the Clinical Research Ethics committee of the Cork Teaching Hospitals (CREC). Patient recruitment into the study was via a Senior Consultant Neurologist and patients had to meet the revised MacDonald diagnostic criteria for clinically defined MS (Polman et al., 2011) including patient history, clinical signs and symptoms, physical examination, and adjunctive diagnostic tools including MRI. The Disability Status Scale scores were taken using the Expanded Disability Status Scale (EDSS) by a Senior Consultant Neurologist in an outpatient clinic. All confirmed MS patients had a RR form of MS as defined by the revised McDonald criteria. Disease severity was scored at time of collection using the Kurtzke’s (1983) EDSS. Patients with RR-MS were clinically stable and naïve to disease modifying therapies including IFN-β, glatiramer acetate, fingolimod, teriflunomide, dimethyl fumarate, natalizumab, and alemtuzumab. PBMCs were collected from venous blood of healthy controls participants (mean age 30.3 ± 1.4 years; *n* = 16) and RR-MS patients (mean age 39.3 ± 3.2 years; *n* = 8) by way of venipuncture as previously described (Downer et al., 2013b). Details of the patient demographics are presented in **Figure 3A**. Human PBMCs were prepared from heparinized venous whole blood samples (50 ml per donor) from individuals by density separation over Lymphoprep^TM^ (Axis-Shield, Norway). PBMCs were plated (1 × 10^6^ cells/ml) on 24-well plates prior to treatment. Plasma samples were separated following centrifugation, aliquoted, and stored at -80°C for subsequent analysis via ELISA.

### Cytokine Analysis in Culture Supernatants

Mixed glia (5 × 10^5^ cells/ml) and PBMCs (1 × 10^6^ cells/ml) were seeded overnight in 24-well plates. Cells were incubated with LPS (100 ng/ml) or Poly(I:C) (10 μg/ml) for timepoints ranging from 10 min–24 h. Mixed glia/PBMCs were also pre-exposed (30 min) to baclofen (10, 30, and 100 μM) prior to LPS (100 ng/ml; 24 h) or Poly(I:C) (10 μg/ml; 24 h) exposure. Supernatants were assayed for tumor necrosis factor-α (TNF-α) and IL-8 concentration by ELISA according to manufacturer’s instructions (Duoset, R&D Systems, Abingdon, UK).

### Quantitative Real-Time PCR

RNA was extracted from PBMCs (untreated) using a NucleoSpin^®^ RNAII isolation kit (Macherey-Nagel Inc., Geschäftsführer, Germany). The concentration of RNA was determined using a UV/V is spectrophotometer. cDNA synthesis was performed on 1 μg RNA using a High Capacity cDNA RT Kit (Applied Biosystems, Carlsbad, CA, USA) according to the manufacturer’s instructions. Equal amounts of cDNA were used for RT-PCR amplification. Real-time PCR primers were delivered as “Taqman^®^ Gene Expression Assays” containing forward and reverse primers, and a FAM-labeled MGB Taqman probe for the target gene (Applied Biosystems). GABA_B1_ primers were used to assess the expression of the target gene (Taqman Gene Expression Assay no. Hs00181306_m1). cDNA (1:4 dilution) was prepared and real-time PCR performed using Applied Biosystems 7300 Real-time PCR System. cDNA was mixed with qPCR^TM^ Mastermix Plus (Applied Biosystems) and the respective gene assay in a 25 μl volume (10 μl of diluted cDNA, 12.5 μl Taqman^®^ Universal PCR Mastermix, 1.25 μl target primer and 1.25 μl 18S). Eukaryotic 18S rRNA was used as an endogenous control and expression was conducted using a gene expression assay containing forward and reverse primers and a VIC-labeled MGB Taqman probe (#4319413E; Applied Biosystems, USA). Samples were run in duplicate and 40 cycles were run as follows: 10 min at 95°C and for each cycle, 15 s at 95°C and 1 min at 60°C. Gene expression was calculated relative to the endogenous control and analysis was performed using the 2^-ΔΔCT^ method. In all experiments no change in relative 18S rRNA expression between treatment groups was observed.

### TNF-α and IL-8 Measurement in Plasma

Plasma samples from healthy donors and RR-MS patients were analyzed for concentrations of TNF-α and IL-8 by ELISA (Duoset, R&D Systems, UK) according to manufacturer’s instructions.

### Immunocytochemistry

Mixed glia (5 × 10^5^ cells/ml) and PBMCs (1 × 10^6^ cells/ml) were seeded overnight on 13-mm diameter coverslips coated with poly-l-lysine (Sigma–Aldrich) and grown for 24 h. Cells were incubated with LPS (100 ng/ml) or Poly(I:C) (10 μg/ml) for timepoints ranging from 10 min–24 h. Mixed glia/PBMCs were also pre-exposed (30 min) to baclofen (10, 30 and 100 μM) prior to LPS (100 ng/ml; 30 min) or Poly(I:C) (10 μg/ml; 6 h) exposure. Cells were then fixed in ice-cold methanol for 10 min, permeabilized with 0.2% Triton X-100 (Thermo Fisher Scientific, Waltham, IL, USA) in PBS for 10 min at room temperature and blocked with 10% goat serum (Vector Laboratories, Peterborough, UK) for 2 h. Cells were treated overnight at 4°C with rabbit polyclonal NF-κB p65 antibody (1:200 in 5% goat serum; Santa Cruz Biotechnology, Santa Cruz, CA, USA). Cells were then washed and incubated with goat anti-rabbit Alexa488 secondary antibody (1:1000 in 5% goat serum; Invitrogen, Dublin, Ireland) and 4′,6-diamidino-2-phenylindole (DAPI; 1.5 μg/ml) in PBS, washed, and mounted (Vectashield; Vector Laboratories). Cells were imaged using an Olympus IX70 inverted microscope. The fluorescence intensity in the nucleus of individual cells stained for NF-κB p65 was measured using the Image J analysis software (Rasband,WJ, http://rsb.info.nih.gov/ij/). The relative fluorescence intensity was calculated as the intensity after subtraction of the background noise. For each treatment, six coverslips were stained and five fields of view were captured per coverslip. Negative control experiments were performed by replacing the primary antibody with PBS and using equal gain settings during acquisition and analysis.

### Statistical Analysis

Data were analyzed using one-way or two-way analysis of variance (ANOVA) as appropriate. When analysis by ANOVA indicated significance (*P* < 0.05), the *post hoc* Student Newman–Keuls test was used. Correlation tests were performed using two-tailed Spearman (non-parametric) correlation coefficient. Data are expressed as means ± SEs of the mean (SEM).

## Results

### TLR4 Activation Time-Dependently Increases Nuclear NF-κB p65 Expression and TNF-α Release in Mixed Glia

**FIGURE 1:**
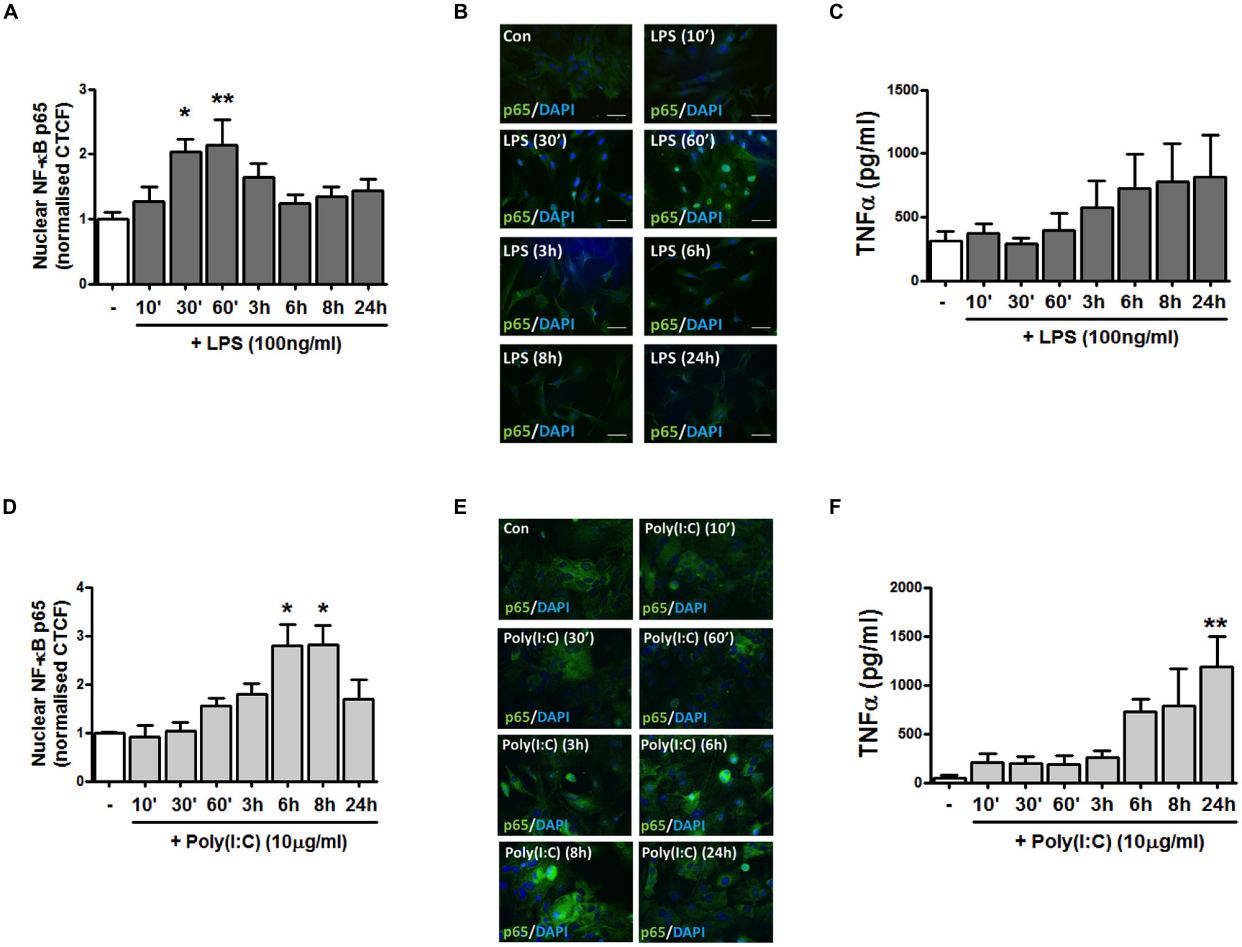
**TLR4 and TLR3 stimulation promotes NF-κB p65 distribution to the nucleus and pro-inflammatory TNF-α expression in primary mixed glial cell cultures. (A,D)** The relative fluorescence intensity (corrected total cell fluorescence; CTCF) of nuclear NF-κB p65 subunit was determined using Image J to assess the subcellular localization of p65 in mixed glial cultures following exposure to **(A)** LPS and **(D)** poly(I:C) for the indicated time periods. **(B,E)** Immunofluorescent images assessing the subcellular localization of p65 in mixed glial cultures following exposure to **(B)** LPS and **(E)** poly(I:C) for the indicated time periods. Nuclei were stained with DAPI, and images obtained using an Olympus IX70 inverted microscope equipped with the appropriate filter sets. Data are presented as the mean ± S.E.M and are representative of six animals. Scale bar = 20μm. Mixed glial cells were incubated with **(C)** LPS and **(F)** poly(I:C) (timepoints from 10 min–24 h) and supernatants were analyzed for TNF-α production using ELISA. Data are presented as the mean ± SEM of triplicate determinations and are representative of six animals. ^∗^*p* < 0.05, ^∗∗^*p* < 0.01 compared with vehicle-treated cells.

TLR4 and TLR3 were initially targeted given their involvement in EAE progression (Touil et al., 2006) and evidence that their expression is dysregulated in MS lesions (Bsibsi et al., 2002). To initially characterize the impact of TLR4 stimulation on pro-inflammatory signaling in primary murine mixed glial cells, we temporally assessed the impact of LPS on the distribution of the NF-κB p65 subunit in response to LPS treatment. Firstly, mixed glia were stimulated with LPS for various timepoints ranging from 10 min–24 h, and the localization of endogenous p65 was assessed by immunofluorescence (**Figures 1A,B**). In vehicle-treated glial cells, p65 is predominantly cytoplasmic, as evidenced by the detection of 488-conjugated immunocomplexes outside of the DAPI-stained regions (**Figure 1B**). Stimulation of glia with LPS time-dependently promoted the accumulation of p65 in the nucleus, peaking at 30 min and 1 h post-LPS exposure (**Figures 1A,B**). We next characterized the impact of TLR4 activation on TNF-α protein production in primary mixed glia. LPS time-dependently enhanced TNF-α expression in primary murine mixed glial cells, with mean maximal stimulatory effects on protein expression observed at 6–24 h (**Figure 1C**), indicating that TLR4 stimulation promotes inflammatory signaling in primary mouse glial cells in our culture system.

### TLR3 Activation Enhances Nuclear NF-κB p65 Expression and TNF-α Release in Mixed Glia

To assess the impact of TLR3 stimulation on inflammatory signaling events in mixed glial cells, we next assessed the effect of poly(I:C) exposure on the distribution of the NF-κB p65 subunit. Primary murine glial cells were incubated with poly(I:C) for timepoints ranging from 10 min–24 h, and the localization of the p65 subunit assessed by immunofluorescence (**Figures 1D,E**). TLR3 stimulation time-dependently promoted the accumulation of NF-κB in the nucleus, peaking at 6 and 8 h post-poly(I:C) exposure (**Figures 1D,E**). This temporal profile differed in response to TLR4 stimulation, where maximal sequestration of NF-κB p65 was observed at 30 min and 1 h post-LPS treatment (**Figure 1A**). Poly(I:C) significantly enhanced TNF-α expression in mixed glial cells, with mean maximal stimulatory effects on protein expression observed at 24 h (**Figure 1F**). This indicates that TLR3 and TLR4 activation in primary glia robustly enhances pro-inflammatory signaling in primary murine glial cells.

### Effect of the GABA_B_ Receptor Agonist Baclofen on TLR4-Induced NF-κB-p65 Nuclear Expression and Pro-Inflammatory Cytokine Release in Mixed Glia

**FIGURE 2:**
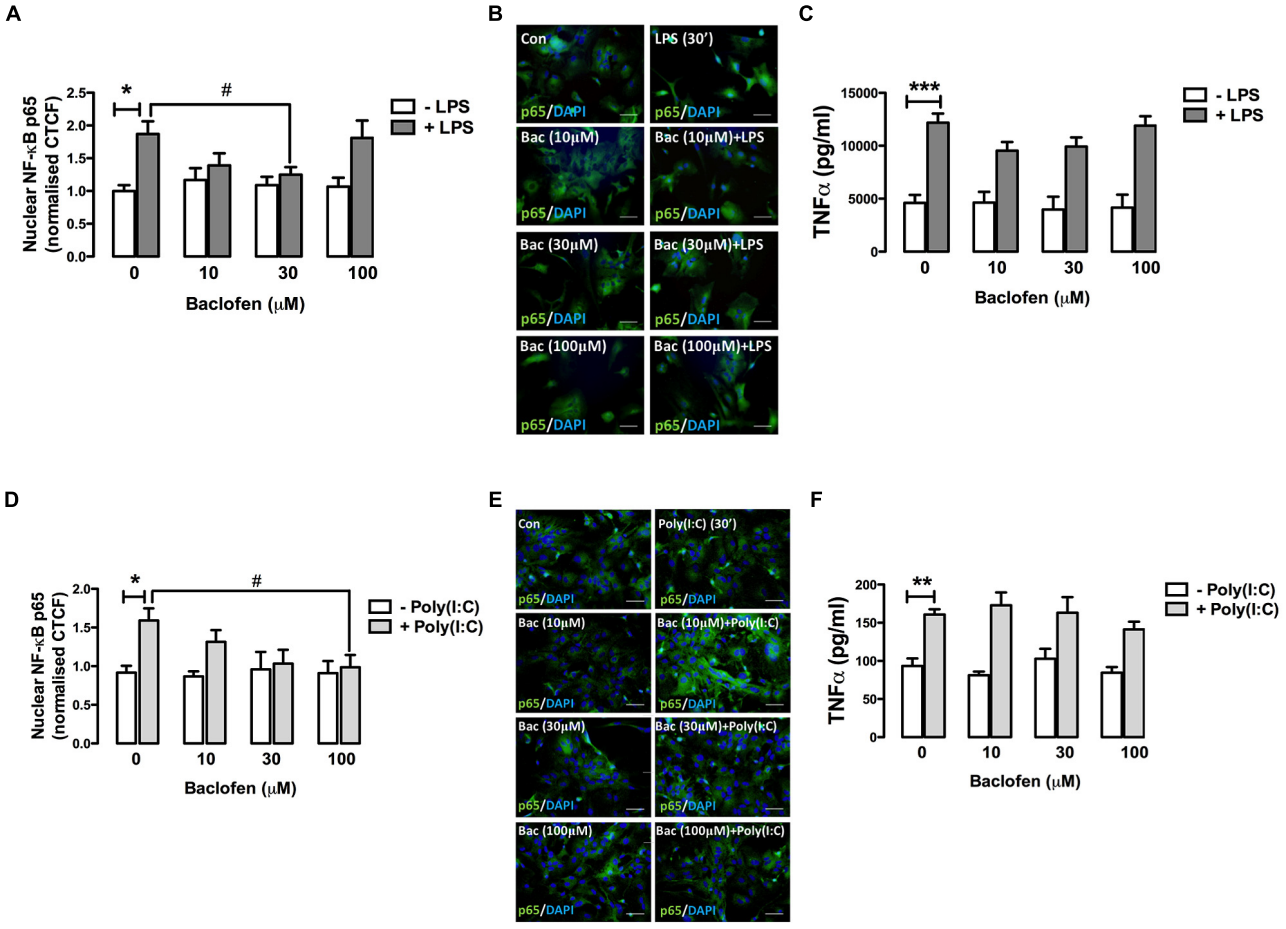
**GABA_B_ receptor activation modulates TLR4- and TLR3-induced NF-κB-p65 nuclear expression and TNF-α expression release in glia. (A,D)** CTCF of nuclear NF-κB p65 subunit in mixed glial cultures following exposure to **(A)** LPS (30 min) and **(D)** poly(I:C) (6 h) in the absence and presence of baclofen (10, 30, and 100 μM). **(B,E)** Immunofluorescent images demonstrating the subcellular localization of p65 in glia following exposure to **(B)** LPS ± baclofen and **(E)** poly(I:C) ± baclofen. Nuclei were stained with DAPI, and images obtained using an Olympus IX70 inverted microscope equipped with the appropriate filter sets. Data are presented as the mean ± SEM and are representative of six animals. Scale bar = 20 μm. Mixed glial cells were pre-incubated with baclofen (10, 30, and 100 μM) prior to **(C)** LPS and **(F)** poly(I:C) (both at 24 h) and supernatants analyzed for TNF-α expression by ELISA. Data are presented as the mean ± SEM of triplicate determinations from six animals. ^∗^*p* < 0.05, ^∗∗^*p* < 0.01, ^∗∗∗^*p* < 0.001 compared with vehicle-treated cells. #*p* < 0.05 compared to cells treated with LPS or Poly(I:C) in the absence of baclofen.

We next examined the impact of the GABA_B_ agonist baclofen on TLR4-induced inflammatory signaling events in primary mixed glia. Glial cultures were pre-treated (30 min) with baclofen (10, 30, and 100 μM) prior to LPS exposure (30 min) and nuclear expression of NF-κB-p65 measured by fluorescence microscopy (**Figures 2A,B**). Stimulation of glia with LPS promoted the accumulation of p65 in the nucleus (**Figure 2A**). Pre-exposure to baclofen (at 30 μM) significantly attenuated the LPS-induced accumulation of p65 in the nucleus, while pre-incubation of glia with the GABA_B_ agonist at concentrations of 10 μM (*p* = 0.09) and 100 μM (*p* = 0.85), failed to significantly impact LPS-induced nuclear p65 sequestration (**Figures 2A,B**). Similarly, **Figure 2C** demonstrates that baclofen attenuated LPS-induced TNF-**α** production in glia at concentrations of 10 and 30 μM, although this did not reach statistical significance. These findings suggest that the proclivity of baclofen to impact TLR4 signaling in primary mixed glia is concentration dependent.

### GABA_B_ Receptor Activation Modulates TLR3-Induced NF-κB-p65 Nuclear Expression and TNF-α Expression in Glia

Given that GABA_B_ activation modulates TLR4-induced signaling in glia, we next assessed the impact of baclofen on TLR3-induced inflammatory signaling in glial cells. Pre-incubation of glia with baclofen dose-dependently attenuated poly(I:C)-induced nuclear sequestration (at 6 h) of NF-κB, reaching significance at 100 μM (**Figures 2D,E**). Baclofen reduced poly(I:C)-induced TNF-**α** production in glia at concentrations of 100 μM, although this did not reach statistical significance (**Figure 2F**). These findings suggest that GABA_B_ activation regulates TLR3 signaling to NF-κB in glia in a concentration-dependent manner.

### Demographic Data of Human Participants

**FIGURE 3:**
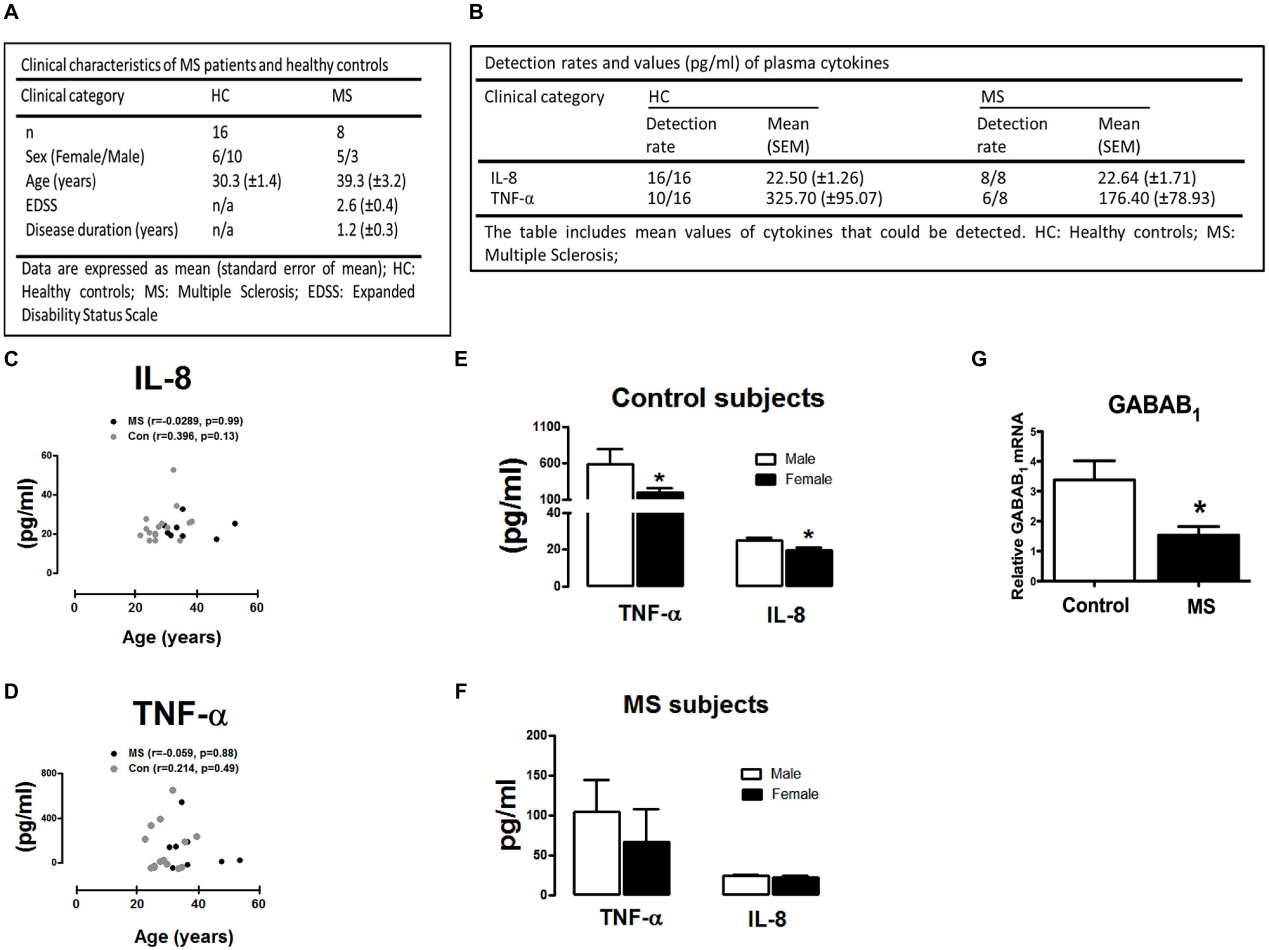
**Demographic and clinical characteristics of MS patients and control subjects included in the study. (A)** Demographics of healthy control participants and RR-MS patients. Expanded Disability Status Scale (EDSS). **(B)** Plasma levels of IL-8 and TNF-α in control (*n* = 16) and MS (*n* = 8) cohort. Detection rates and mean plasma expression values of IL-8 and TNF-α are summarized. **(C,D)** Correlation between IL-8/TNF-α plasma levels and age of study participants. Correlation plots demonstrating **(C)** IL-8 and **(D)** TNF-α plotted against subject age. No correlation was observed between age in control and MS groups analyzed (two-tailed Spearman non-parametric correlation coefficient, correlation *r*-values, and significance *p*-values, are indicated in the graphs). Levels of TNF-α and IL-8 in **(E)** healthy control participants and **(F)** MS patients in plasma obtained from male (*n* = 13) and female (*n* = 11) participants. **(G)** Peripheral blood mononuclear cells (PBMCs) isolated from healthy subjects and newly diagnosed RR-MS patients were assessed for GABA_B_ mRNA expression, cDNA generated and assayed by quantitative real-time PCR for levels of GABAB_1_ mRNA. Data are presented as the mean ± SEM of triplicate determinations. ^∗^*p* < 0.05 compared with male control subjects **(E)**. ^∗^*p* < 0.05 compared with all control subjects **(G)**.

A total of 24 subjects were assessed consisting of healthy control participants (*n* = 16) and newly diagnosed RR-MS patients (*n* = 8). All RR-MS patients were drug naïve at the time of inclusion. Mean disease duration in the RR-MS cohort was 1.2 ± 0.3 years and subjects had mild to moderate disability as reflected by EDSS scores of 2.6 ± 0.4 on average. The mean age of the 24 participants who enrolled in the investigation was 33.1 ± 7.8 years (range 23–40 years in control cohort; range 31–54 years in RR-MS cohort) and 46% of the sample was female. The overall demographics of the participants are reported in **Figure 3A**.

### Cytokine and Chemokine Analysis in Human Plasma

To determine if RR-MS is associated with alterations in peripheral inflammatory signature, we initially assessed plasma levels of the inflammatory chemokine IL-8 and cytokine TNF-α in plasma from control and RR-MS groups. No significant difference in plasma IL-8 (*p* = 0.94) and TNF-α (*p* = 0.28) concentration was found between groups (**Figure 3B**). Detection rates and mean values of plasma TNF-α and IL-8 are summarized in **Figure 3B**. In addition, no clear correlation between the levels of plasma cytokines and participant age was determined in either RR-MS patient or control groups (two-tailed Spearman non-parametric correlation coefficient; **Figures 3C,D**), indicating that participant age had no impact on peripheral cytokine/chemokine expression. We next assessed the impact of gender on plasma cytokine and chemokine levels. Interestingly, compared to male control subjects, the expression levels of TNF-α and IL-8 were significantly reduced in female control subjects (**Figure 3E**). No significant difference was determined between males vs. females in the RR-MS cohort (**Figure 3F**). This data indicates that the level of cytokine/chemokine expression in healthy individuals is higher in males than females, but that this gender difference is not apparent in the RR-MS cohort.

### GABA_B_ Expression in Human PBMCs

To determine the expression profile of the metabotropic receptor GABA_B_ in human PBMCs, and whether this profile was altered in disease, we assessed the expression of GABA_B1_ subunit in PBMCs isolated from healthy volunteers and RR-MS patients. Detectable levels of the GABA_B1_ subunit was determined in human PBMCs, with RR-MS associated with a reduction (twofold) in endogenous GABA_B1_ expression when compared to PBMCs isolated from healthy subjects (**Figure 3G**). This indicates that downregulation of GABA_B_ receptors occurs in PBMCs isolated from newly diagnosed RR-MS patients, when compared to immune cells from healthy subjects.

### Effect of TLR3 and TLR4 Stimulation on PBMCs Isolated from Control and RR-MS Cohorts

**FIGURE 4:**
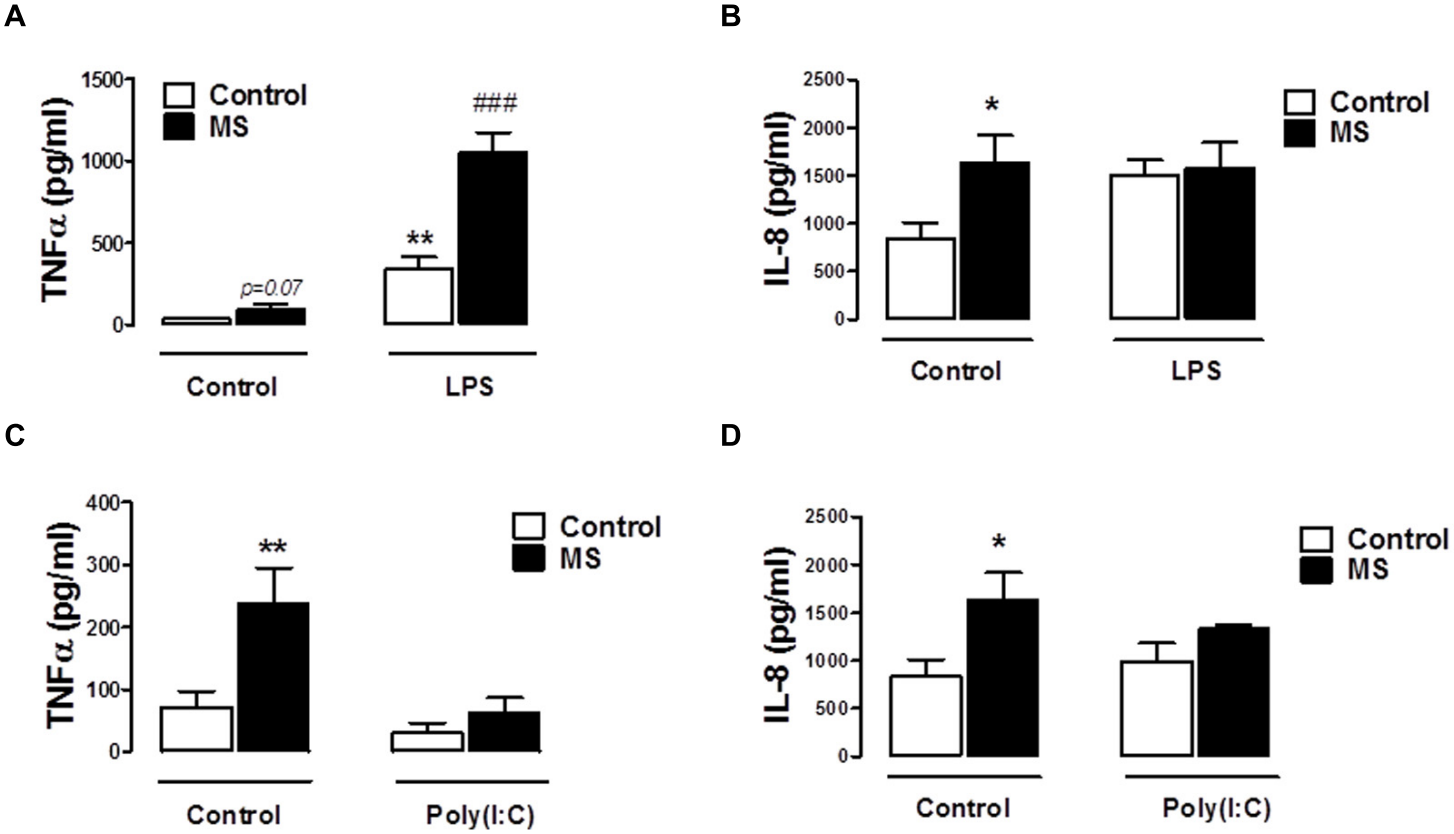
**Peripheral blood mononuclear cells from RR-MS patients display enhanced inflammatory signaling.** PBMCs prepared from healthy subjects and MS patients were seeded into 24-well plates and treated with vehicle, LPS (100 ng/ml) or poly(I⋅C) (10 μg/ml) for 24 h. Supernatants were analyzed for TNF-α **(A,C)** and IL-8 **(B,D)** production using ELISA. Data are presented as the mean ± SEM of triplicate determinations. ^∗^*p* < 0.05 and ^∗∗^*p* < 0.01 compared with vehicle-treated PBMCs from healthy subjects. ^###^*p* < 0.001 compared with vehicle-treated cells from MS patients.

Given the defined role of TLR3 and TLR4 in EAE progression (Touil et al., 2006), in addition to evidence that their expression is up-regulated in MS lesions (Bsibsi et al., 2002), we next set out to assess the impact of TLR3 and TLR4 stimulation on inflammatory responses of immune cells isolated from control and RR-MS patients. Firstly, supernatants from unstimulated PBMCs isolated from RR-MS patients displayed enhanced TNF-α (**Figures 4A,C**) and IL-8 (**Figures 4B,D**) expression, when compared to PBMCs from healthy subjects, indicating the RR-MS patient PBMCs display an enhanced endogenous inflammatory signature. LPS enhanced TNF-α production in PBMCs from both groups (**Figure 4A**), and importantly the production of TNF-α was exacerbated in PBMCs prepared from the RR-MS group, compared with the control cohort (**Figure 4A**). In terms of IL-8 expression, two-way ANOVA revealed a significant influence of disease status on IL-8 levels [*F*_(1,30)_ = 4.773, *p* = 0.0369], but no significant influence of TLR4 stimulation on overall variation [*F*_(2,30)_ = 0.7174, *p* = 0.4962] (**Figure 4B**). However, *post hoc* analysis revealed a trend toward a statistically significant increase in IL-8 release following LPS treatment in PBMCs isolated from healthy controls (*p* = 0.0612), but not in RR-MS patients. Interestingly, PBMCs isolated from healthy subjects and RR-MS patients were unresponsive to poly(I⋅C) stimulation, both in terms of TNF-α and IL-8 production (**Figures 4C,D**), indicating that PBMCs from control individuals and RR-MS patients are refractory to TLR3 stimulation, in terms of TNF-α and IL-8 signaling.

### Baclofen Attenuates TLR4-Induced TNF-α Cytokine Release in Human PBMCs Isolated from Healthy, but not RR-MS, Subjects

**FIGURE 5:**
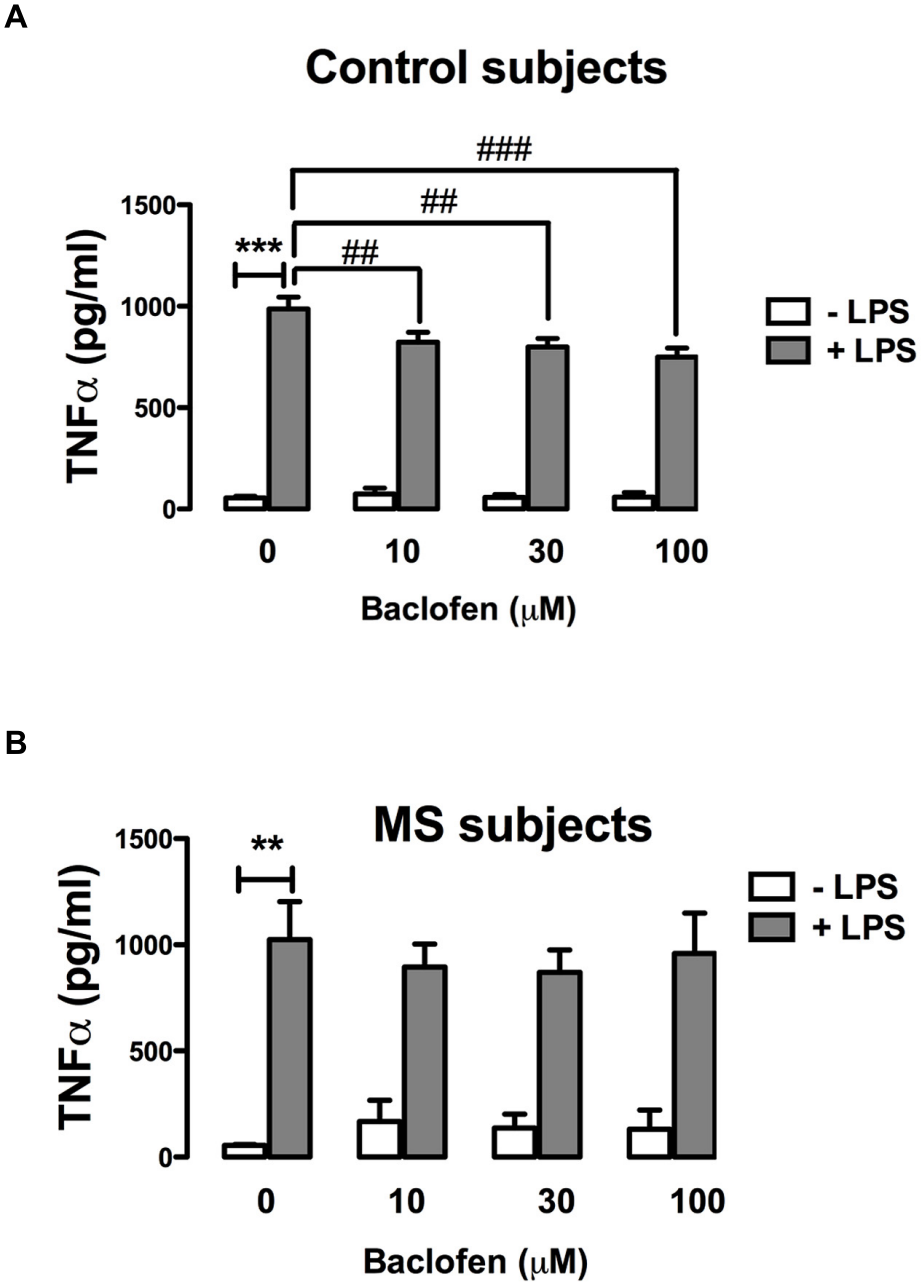
**GABA_B_ activation attenuates TLR4-induced TNF-α production in PBMCs from healthy, but not MS, subjects.** PBMCs isolated from **(A)** healthy subjects and **(B)** MS patients were seeded in 24-well plates and pre-treated with baclofen (10, 30, and 100 μM) in the absence and presence of LPS (100 ng/ml) for 24 h. Supernatants were analyzed for TNF-α production using ELISA. Data are presented as the mean ± SEM of triplicate determinations. ^∗∗^*p* < 0.01 and ^∗∗∗^*p* < 0.001 compared with vehicle-treated cells. ^##^*p* < 0.01 and ^###^*p* < 0.001 compared to cells treated with LPS in the absence of baclofen.

Given that GABA_B_ activation modulates TLR4-induced signaling in primary mixed glia (**Figure 2**), and that TLR4 stimulation enhances TNF-α signaling in PBMCs from control and RR-MS patients (**Figure 4**), we next assessed the proclivity of baclofen to modulate TLR4-induced TNF-α release in PBMCs isolated from healthy subjects and RR-MS patients. PBMCs from healthy subjects (**Figure 5A**) and RR-MS patients (**Figure 5B**) were pre-treated (30 min) with baclofen (10, 30, and 100 μM) prior to LPS exposure (24 h) and TNF-α expression assessed. PBMCs isolated from healthy subjects were responsive to LPS with an increase in TNF-α release observed, whereas baclofen modestly attenuated this (**Figure 5A**). In contrast, pre-exposure (30 min) to baclofen prior to LPS exposure (24 h) failed to significantly impact TLR4-induced TNF-α expression in PBMCs from RR-MS patients at each dose tested (**Figure 5B**). Importantly, this indicates a differential sensitivity of cells from healthy and RR-MS subjects in terms of the ability of baclofen to modulate TLR-induced inflammatory signaling, which may have implications in terms of the design of GABA_B_ receptor therapeutics to treat neuroinflammatory disorders including MS.

## Discussion

Understanding the mechanisms underlying neuroinflammatory processes in MS may pave the way to novel therapeutic strategies. Data herein demonstrate that the GABA_B_ receptor agonist baclofen has the proclivity to differentially target TLR4 and TLR3 inflammatory signaling events both in CNS glial cells, and in peripheral immune cells. Baclofen reduced TLR3/4-induced nuclear NF-κB and TNF-α (albeit modestly) expression in glial cells, with distinct dose-dependency identified for these effects. GABA_B_ receptor activation attenuated TLR4-induced TNF-α expression in PBMCs isolated from healthy individuals, while baclofen had no impact on TLR4 signaling in RR-MS patient PBMCs. Furthermore, PBMCs from RR-MS patients expressed lower levels of the GABA_B_ receptor mRNA. Significantly, we also determined that immune cells from the RR-MS patient group displayed hypersensitivity to LPS exposure in terms of TNF-α expression.

TLR family members are expressed in cells of the CNS (Downer et al., 2013a; Nakano et al., 2015) and TLR ligands regulate CNS inflammation (Lehnardt, 2010). We initially targeted TLR3 and TLR4 signaling given the role of these receptors in neuroinflammatory events linked with MS (Bsibsi et al., 2002; Touil et al., 2006). Both NF-κB activation and pro-inflammatory cytokine expression are known downstream signaling events following ligation of TLR3 and TLR4 (Moynagh, 2005), and signaling via both cascades is perturbed following TLR3 (Cavassani et al., 2008) and TLR4 (Devaraj et al., 2011) knockout. We examined the ability of LPS and poly(I:C) to couple to TLR4 and TLR3 inflammatory signaling in primary glia, respectively. Data presented herein indicate that LPS and poly(I:C) enhanced NF-κB activation and TNF-α cytokine expression in glia, with the kinetics of this response mirroring data described elsewhere (Park et al., 2006; Horvath et al., 2008).

This study highlights the anti-inflammatory potential of baclofen in glial cells, by virtue of its inhibitory effects on the NF-κB-TNF-α pathway induced by TLR3/4 activation. This is consistent with data elsewhere indicating that baclofen reduces LPS-induced production of IL-6/IL-12 in mouse microglia (Kuhn et al., 2004), and reduces phospho-p65-NF-κB and IL-6/TNF-α expression in human astrocytes and microglial cells stimulated with IFN-γ/LPS (Lee et al., 2011). GABA_B_ receptors are expressed on astrocytes and microglia (Charles et al., 2003), and evidence indicates that glial GABA_B_ receptors are functional (Kuhn et al., 2004; Oka et al., 2006). Given the expression and functional activity of GABA_B_ receptors on glia, alongside evidence that baclofen negatively regulates TLR3/4-induced inflammatory signaling, it is likely that GABA_B_ receptors play an integral role in the course of neuroinflammation by targeting glia. The ability of baclofen to target TLR3/4-induced inflammatory signaling in glia *in vitro* may contribute to an anti-inflammatory efficacy *in vivo*, and this will be the focus of further studies.

Interestingly, different effects of GABA_B_ receptor modulation on the immune system have been indicated, suggesting that GABA is a regulator of immune cell activity and inflammation. Indeed, GABA receptors have been identified on neutrophils, macrophages, dendritic cells, and T-cells (Rane et al., 2005; Jin et al., 2013), and cells of the immune system possess the metabolic machinery for the synthesis of GABA (Auteri et al., 2015). Given the evidence that GABA is a neuroimmune modulator, alongside our findings that baclofen modulates TLR-induced inflammatory signaling in central glial cells, we next assessed the impact of baclofen on TLR signaling in peripheral immune cells. Firstly, our findings indicate that TLR4 activation promotes TNF-α and IL-8 cytokine expression in human PBMCs, and these findings are consistent with data elsewhere (Kaplin et al., 2009; Downer et al., 2013b). Importantly, data presented herein indicate an anti-inflammatory propensity of baclofen in human immune cells, demonstrating that baclofen blunts LPS-induced TNF-α expression in peripheral immune cells from healthy subjects. This finding is in line with data demonstrating that exposure of human PBMCs to baclofen ameliorates phytohemagglutinin-induced TNF-α release (Duthey et al., 2010). A significant finding here is that this is the first direct evidence that baclofen can target TLR signaling in human PBMCs, and hence has relevance across a range of neuroimmune disorders.

Our results identify that PBMCs isolated from healthy subjects were responsive to baclofen, with a significant, albeit moderate, decrease in LPS-induced TNF-α expression observed following baclofen treatment. In contrast, baclofen failed to regulate TLR4-induced TNF-α expression in PBMCs from RR-MS subjects. This is significant as intrathecal administration of baclofen can be used to treat spasticity in MS, in addition to spinal cord injury, cerebral palsy, and acquired brain injury (Gunnarsson and Samuelsson, 2015). Furthermore, there is evidence of a loss of GABAergic neurons at lesion sites in post-mortem MS brain (Redondo et al., 2014). The differential sensitivity of cells from healthy and RR-MS subjects to baclofen indicates that MS drives a desensitizing signal in terms of GABA_B_ receptor function. Indeed, our findings indicate that downregulation of GABA_B_ receptors occurs in PBMCs in newly diagnosed RR-MS patients, which may govern the differential sensitivity to baclofen exposure seen in healthy and MS patient cells. In support of this, the level of GABA is reduced in the blood serum of patients with MS as compared to the controls (Demakova et al., 2003). In addition, baclofen treatment has been shown to reduce GABA_B_ receptor density in rat spinal cord (Kroin et al., 1993), which may be postulated as a molecular mechanism for development of tolerance. It is especially interesting to note that treatment failure has been shown with baclofen in MS patients (Stroet et al., 2013), and the inability of baclofen to target TLR4-induced pro-inflammatory cytokine production might contribute to these effects.

Whether the effects of baclofen identified in the present and other studies are a direct result of its action at the GABA_B_ receptor is not yet entirely clear, particularly given the recent discovery that baclofen, and several GABA_B_ receptor antagonists, are allosteric modulators of CXCR4, a chemokine receptor involved in neuroimmune crosstalk (Guyon et al., 2013). Indeed, determining whether the effects of baclofen that were observed in this study are specifically due to its action at the GABA_B_ receptor rather than at CXCR4 will be the focus of future studies. Such studies will be challenging because several GABA_B_ receptor antagonists also act as allosteric modulators of CXCR4 (Guyon et al., 2013). Future studies aimed at identifying GABA_B_ receptor antagonists that do not affect CXCR4 activity will be required in order to address this question directly. Nevertheless, since baclofen and some GABA_B_ receptor antagonists exert the same effects on CXCR4 activity (Guyon et al., 2013), then it would be expected that prevention of the effects of baclofen by a GABA_B_ receptor antagonist would be independent of CXCR4 activity (since both baclofen and the GABA_B_ receptor antagonists behave as CXCR4 antagonists). Alternatively, experiments could examine the effects of baclofen under conditions whereby CXCR4 expression or activity is altered, however, such experiments might be complicated by the modulation of inflammatory mediators by CXCR4 itself. Despite these limitations, there is accumulating evidence that many components of the GABA neurotransmitter system, including not only the GABA_B_ receptor but also the GABA_A_ receptor, can have anti-inflammatory effects (Song et al., 1998; Bhat et al., 2010; Lee et al., 2011).

The rationale behind the present study was to determine if baclofen could target TLR-induced signaling in RR-MS, particularly given the growing body of data indicating that the TLR system is a key player in MS pathogenesis (O’Brien et al., 2008; Downer, 2011). However, intrathecal administration of baclofen is used to manage spasticity in patients with a range of conditions including spinal cord injury, cerebral palsy and acquired brain injury in addition to MS, and hence in future studies it would be interesting to compare the effects of baclofen on the PBMCs of patients with other neurological conditions.

The concentrations of cytokines/chemokines are altered in MS (Ubogu et al., 2006), suggesting that cytokine/chemokine signatures may indicate disease progression in patient groups (O’Connell et al., 2014). Given that evidence indicates that TNF-α (Sharief and Hentges, 1991) and IL-8 (Lund et al., 2004) are higher in serum of patients with MS, we examined the relative expression of TNF-α and IL-8 in plasma isolated from newly diagnosed RR-MS patients. Our analysis revealed no difference in plasma IL-8/TNF-α between control and RR-MS groups. Systemic inflammation in MS is strongly associated with periods of intense relapse (Confavreux et al., 2000), hence it is plausible that alterations in peripheral cytokines in MS may be phase dependent. In support of this, Mellergard et al. (2010) indicate that MS patients with EDSS measurements in the range comparable to the RR-MS cohort assessed in present study demonstrate no alteration in plasma TNF-α when compared to healthy subjects (Mellergard et al., 2010).

Neuroinflammatory changes develop with age, with defects in the immune system identified in elderly individuals (Allman and Miller, 2005). In addition, an increased plasma level of pro-inflammatory cytokines has been demonstrated in elderly, compared to young, individuals (Forsey et al., 2003). Our findings indicate no clear correlation between the levels of plasma cytokine/chemokine and participant age in either RR-MS or control groups, indicating that the age of subjects did not impact cytokine/chemokine profiles. Interestingly, compared to male control subjects, the expression levels of TNF-α and IL-8 were significantly reduced in female control subjects. This indicates that particular expression profiles of cytokines/chemokines may be gender specific, which is confirmed elsewhere (Asai et al., 2001).

Marked alterations in the expression profile of TLRs has been determined in MS lesions in human brain samples, in the brain samples from mice that have undergone EAE, and in CSF cells isolated from MS patients (Marta, 2009). With this in mind we investigated the effect of TLR4 and TLR3 stimulation on the pro-inflammatory cytokine and chemokine, TNF-α and IL-8. PBMCs without stimulation, as well as after stimulation with LPS, displayed increased production of TNF-α and IL-8 in the RR-MS sample cohort, when compared to the control group. These results suggest that immune cells from the RR-MS patient group have an enhanced endogenous inflammatory signature. In addition, RR-MS patient PBMCs displayed hypersensitivity to LPS exposure in terms of TNF-α expression. TLR4 expression is upregulated in CNS lesions in mice following EAE and in PBMCs from RR-MS and secondary progressive (SP) MS patients (Andersson et al., 2008), which in support of our study, indicates that TLR4 signaling participates in an innate immune response that may shape the inflammatory response in both forms of MS. It is intriguing that the hypersensitivity of RR-MS patient cells to LPS is only relevant in the context of TNF-α induction, since LPS shows comparable efficacy in inducing IL-8 in cells from healthy and RR-MS patients. Hence, any form of TLR4 hypersensitivity that may exist in MS patient cells appears to be restricted to the pathway leading to TNF-α expression, and further studies will probe the intracellular signaling events in patient cells.

Our studies also probed the effects of TLR3 stimulation on TNF-α/IL-8 in PBMCs. Surprisingly, PBMCs isolated from healthy donors and RR-MS patients did not respond to TLR3 stimulation by enhancing TNF-α or IL-8 expression, suggesting that the TLR3 pathway leading to cytokine and chemokine expression may be desensitized in our participant cohorts. Indeed, we have previously demonstrated that the TLR3-IFN-β pathway is desensitized in MS patient PBMCs, suggesting that MS patients may be pre-sensitized to viral infection showing some form of TLR3 tolerance. However, the non-responsiveness of healthy donor PBMCs to poly(I⋅C) is in line (Wesch et al., 2006) and in contrast (Myles et al., 2012) to findings elsewhere in studies which investigated freshly isolated PBMCs; differences in dose and timecourse for treatment regimen used with poly(I:C) in the present study may underlie the differences observed.

Although baclofen demonstrates therapeutic effects in MS, the mechanism(s) of action are poorly understood. We present evidence that the innate arm of the immune system is a target for baclofen anti-inflammatory action, and demonstrate that baclofen differentially targets TLR4 and TLR3 inflammatory signaling events both in primary murine glial cells and in PBMCs isolated from human blood samples. Baclofen can exert anti-inflammatory properties at specific doses, by down-regulating TLR-induced activation of NF-κB and induction of pro-inflammatory cytokines. Significantly, our findings also indicate that inflammatory signaling, and sensitivity to TLR4 stimulation, was enhanced in PBMCs of patients with RR-MS, highlighting that TLR4 may play a role at least in the RR form of MS pathogenesis. We targeted this particular patient cohort because they were newly-diagnosed and naïve to disease modifying therapies. However, investigating TLR functioning and its modulation by baclofen in patients with secondary chronic progressive MS would be an important future study. Overall, the present study provides novel insight into the cellular effects of targeting central and peripheral GABA_B_ receptors in the modulation of TLR functioning.

## Conflict of Interest Statement

The authors declare that the research was conducted in the absence of any commercial or financial relationships that could be construed as a potential conflict of interest.
