# Supplementary figures and images for: Modulation of TLR3/TLR4 inflammatory signaling by the GABAB receptor agonist baclofen in glia and immune cells: relevance to therapeutic effects in multiple sclerosis
Source: Front Cell Neurosci. 2015 Jul 28;9:284. doi: 10.3389/fncel.2015.00284 (PMC4516894; doi:10.3389/fncel.2015.00284)

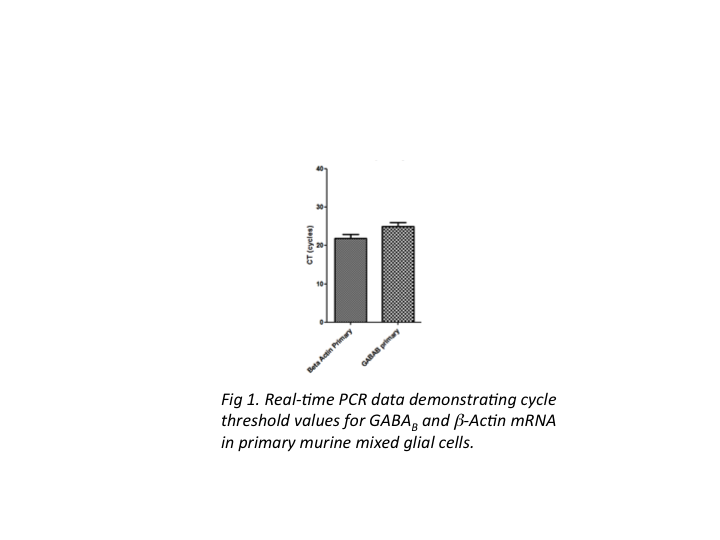

Supplement: Supplementary file 1 [file Image_1.TIFF]
